# Sarcopenia as an Independent Risk Factor for Specific Cancers: A Propensity Score-Matched Asian Population-Based Cohort Study

**DOI:** 10.3390/nu14091910

**Published:** 2022-05-02

**Authors:** Ming-Yang Sun, Chia-Lun Chang, Chang-Yun Lu, Szu-Yuan Wu, Jia-Qiang Zhang

**Affiliations:** 1 Department of Anesthesiology and Perioperative Medicine, People’s Hospital of Zhengzhou University, Henan Provincial People’s Hospital, Zhengzhou 450052, China; jqzhang@henu.edu.cn; 2 Department of Hemato-Oncology, Wan Fang Hospital, Taipei Medical University, Taipei 110, Taiwan; 101255@w.tmu.edu.tw; 3 Department of Internal Medicine, School of Medicine, College of Medicine, Taipei Medical University, Taipei 110, Taiwan; 4 Department of General Surgery, Lo-Hsu Medical Foundation, Lotung Poh-Ai Hospital, Yilan 265, Taiwan; 92115@w.tmu.edu.tw; 5 Department of Food Nutrition and Health Biotechnology, College of Medical and Health Science, Asia University, Taichung 413, Taiwan; 6 Big Data Center, Lo-Hsu Medical Foundation, Lotung Poh-Ai Hospital, Yilan 265, Taiwan; 7 Division of Radiation Oncology, Lo-Hsu Medical Foundation, Lotung Poh-Ai Hospital, Yilan 265, Taiwan; 8 Department of Healthcare Administration, College of Medical and Health Science, Asia University, Taichung 413, Taiwan; 9 Graduate Institute of Business Administration, Fu Jen Catholic University, Taipei 242, Taiwan; 10 Centers for Regional Anesthesia and Pain Medicine, Wan Fang Hospital, Taipei Medical University, Taipei 110, Taiwan

**Keywords:** sarcopenia, cancers risk, IRRs, survival

## Abstract

**Simple Summary:**

Whether preexisting sarcopenia independently leads to cancer incidence remains unclear. Our study investigated the effect of sarcopenia on cancer incidence. We designed a propensity score–matched population-based cohort study to demonstrate that sarcopenia onset before cancer might be associated with cancer risk. We determined the significant adjusted incidence rate ratios (IRRs) of cancer risk on lung, colorectal, breast, head and neck, pancreas, gastric, esophageal, and ovarian cancers and hepatocellular carcinoma in patients with or without diagnosed sarcopenia before cancer.

**Abstract:**

Purpose: Whether preexisting sarcopenia is an independent risk factor for cancer incidence remains unclear. Therefore, we performed this propensity score (PS)-matched (PSM) population-based cohort study to compare the incidence rate ratios (IRRs) of specific cancers between patients with and without sarcopenia. Patients and Methods: The patients were categorized into two groups according to the presence or absence of sarcopenia, matched at a 4:1 ratio. Results: PS matching yielded a final cohort of 77,608 patients (15,527 in the sarcopenia and 62,081 nonsarcopenia groups) eligible for further analysis. In our multivariate Cox regression analysis, compared with the nonsarcopenia group, the adjusted hazard ratio (aHR; 95% confidence interval (CI)) for cancer risk in the sarcopenia group was 1.277 (1.10 to 1.36; *p* < 0.001). Furthermore, the adjusted IRRs (95% CIs) for sarcopenia patients were pancreatic cancer 3.77 (1.79 to 4.01), esophageal cancer 3.38 (1.87 to 4.11), lung cancer 2.66 (1.15 to 2.90), gastric cancer 2.25 (1.54 to 3.23), head and neck cancer 2.15 (1.44 to 2.53), colorectal cancer 2.04 (1.77 to 2.30), hepatocellular carcinoma 1.84 (1.30 to 2.36), breast cancer 1.56 (1.12 to 1.95), and ovarian cancer 1.43 (1.10 to 2.29), respectively. Conclusions: Sarcopenia might be a significant cancer risk factor for lung, colorectal, breast, head and neck, pancreas, gastric, esophageal, and ovarian cancer, as well as hepatocellular carcinoma.

## 1. Introduction

Sarcopenia, a syndrome characterized by the loss of muscle mass, strength, and performance [1], can occur in not only overweight and underweight individuals but also those with normal weight [2]. Moreover, in a study, 53–57% of older men and 43–60% of older women were diagnosed as having sarcopenia [3]. In contrast to cachexia, sarcopenia does not require the presence of an underlying illness [4]. In addition, although most people with cachexia are sarcopenic, most individuals with sarcopenia are not considered cachectic [4]. Sarcopenia is associated with increased functional impairment, disability, fall, and mortality rates [2]. The causes of sarcopenia are multifactorial, and they can include disuse, endocrine function alteration, chronic diseases, inflammation, insulin resistance, and nutritional deficiencies [1].

Sarcopenia has been found to be associated with increased mortality among patients with cancer [5,6,7]. Specifically, a study found sarcopenia to be significantly associated with mortality for most cancers, except hormone-related cancers (endometrial, breast, ovarian, and prostate cancers) and hematopoietic cancers [8]. Therefore, sarcopenia may be a major prognostic factor for mortality in patients with cancer [5,6,7,8]. Sarcopenia-related cancer mortality might also be a consequence of treatment-related toxicity [9]. However, although sarcopenia has been concluded to be an independent prognostic factor for poor overall survival in patients with cancer [5,6,7,8], whether it is an independent risk factor for cancer remains unclear, and which cancer types are attributable to sarcopenia also remain unknown.

Because sarcopenia is preventable and treatable, understanding the association of sarcopenia with cancer risk is essential [10]. Specifically, establishing whether the alleviation of sarcopenia leads to cancer risk reduction can have major future implications in the field of preventive medicine. Therefore, the current study focused on determining whether preexisting sarcopenia is an independent risk factor for cancer incidence. We used the cohort data from the Taiwan’s National Health Insurance (NHI) Research Database (NHIRD) as the study data. Taiwan’s NHIRD contains all of the registration files and details about the original claims data of all NHI beneficiaries (i.e., approximately 27.38 million individuals). All NHIRD data—which are encrypted to protect the beneficiaries’ privacy—include the detailed outpatient and inpatient claims data, including patient identification number; birth date; sex; diagnostic codes according to the International Classification of Diseases, Ninth Revision, Clinical Modification (ICD-9-CM), and International Classification of Diseases, Tenth Revision, Clinical Modification (ICD-10-CM); treatment information; medical cost; dates of hospital admission and discharge; and date of death [11,12,13]. This was the first and largest comparative propensity score (PS) matching (PSM)-based study, mimicking a case control study, on the association of sarcopenia with cancer risk, including patients with and without sarcopenia.

## 2. Patients and Methods

### 2.1. Data Sources and Study Cohort

We used the January 2008–December 2019 data from the Taiwan NHIRD. All data sets can be interlinked through patient identification numbers. The study protocols were reviewed and approved by the Institutional Review Board of Tzu-Chi Medical Foundation (IRB109-015-B).

### 2.2. Participant Selection

In the main study cohort, we initially enrolled 15,527 NHI beneficiaries diagnosed as having sarcopenia and 88,398 NHI beneficiaries not diagnosed as having sarcopenia over 1 January 2008–31 December 2019 into a sarcopenia and a nonsarcopenia group, respectively.

Before 20026, there was no consensus on the definition of sarcopenia, a variety of diagnostic criteria are being used [14]. In October 2016, the US Centers for Disease Control and Prevention formally recognized sarcopenia as a disease, coding it as M62.84 in IC.

D-10-CM [15]: In general, the sarcopenia-related ICD-9-CM codes 728.2 and 728.9 can be considered equivalent to the ICD-10-CM code M62.84 [16]. The criteria have been used by other studies and are considered to be similar to a diagnosis of sarcopenia [16]. In addition, the diagnosis of the sarcopenia-related ICD-9-CM codes 728.2 and 728.9 and ICD-10-CM code M62.84 were all verified by the professional specialists (such as rehabilitation, orthopedic, or family physician). We defined the sarcopenia group in our study as “sarcopenia, muscular wasting, disuse atrophy, and disorder”.

The index date was defined as the date of sarcopenia onset. Patients diagnosed as having sarcopenia after cancer diagnosis (except for cancer treatment–related sarcopenia) and those with sarcopenia diagnosed within 2 years before cancer diagnosis were excluded. The endpoint was a cancer diagnosis in patients with sarcopenia (vs. controls (i.e., patients without sarcopenia)).

### 2.3. PSM and Covariates

After adjustments for confounders, a Cox proportional hazards model was used to calculate the time from the index date to cancer development in patients with and without sarcopenia. To reduce the effects of potential confounders when comparing cancer risk between the sarcopenia and nonsarcopenia groups, all patients were PS-matched by using the following variables: age, sex, Charlson comorbidity index (CCI) Scores, diabetes, hypertension, hyperlipidemia, tuberculosis, pneumoconiosis, upper respiratory tract infection, hepatitis B, hepatitis C, liver cirrhosis, inflammatory bowel disease, familial adenomatous polyposis, urinary tract infection, Parkinson’s disease, child delivery, gum and periodontal disease, gastric or duodenal ulcer, sleep disorder, alcohol habits, cigarette smoking, income levels, and urbanization (Table 1).

Repeated comorbidities were excluded from the CCI scores to prevent repetitive adjustment in multivariate analysis. Comorbidities were determined according to ICD-9-CM in the main diagnosis of inpatient records or if the number of outpatient visits was ≥2 within 1 year. Comorbidities that presented 2 years before the date of cancer diagnosis were recorded.

Here, continuous variables are presented as means ± standard deviations, where appropriate. We matched participants at a ratio of 4:1 by using the greedy method, with age, sex, CCI scores, diabetes, hypertension, hyperlipidemia, tuberculosis, pneumoconiosis, upper respiratory tract infection, hepatitis B, hepatitis C, liver cirrhosis, inflammatory bowel disease, familial adenomatous polyposis, urinary tract infection, Parkinson’s disease, child delivery, gum and periodontal disease, gastric or duodenal ulcer, sleep disorder, alcohol habits, cigarette smoking, income levels, and urbanization—all with a propensity score within a caliper of 0.2 [17]. Matching is a common technique used for selecting controls with identical background covariates as study participants so as to minimize differences among the study patients (that we deem necessary to be controlled). A Cox model was used to perform the regression of cancer risk variables in patients with and without sarcopenia, and a robust sandwich estimator was used to account for clustering within matched sets [17]. A multivariate Cox regression analysis was performed to calculate hazard ratios (HRs) with 95% confidence intervals (CIs) for determining whether variables listed in Table 1 are the potential independent predictors for cancer risk.

### 2.4. Incidence Rate and Incidence Rate Ratios

The primary endpoint was the incidence rate ratio (IRR) with 95% CI for the sarcopenia versus nonsarcopenia groups calculated by using Poisson regression, with 1000 person-years as an offset, as well as adjustments for age, sex, CCI scores, diabetes, hypertension, hyperlipidemia, tuberculosis, pneumoconiosis, upper respiratory tract infection, hepatitis B, hepatitis C, liver cirrhosis, inflammatory bowel disease, familial adenomatous polyposis, urinary tract infection, Parkinson’s disease, child delivery, gum and periodontal disease, gastric or duodenal ulcer, sleep disorder, alcohol habits, cigarette smoking, income levels, and urbanization.

### 2.5. Statistical Analysis

All statistical analyses were performed on SAS (version 9.4; SAS Institute, Cary, NC, USA). The PSM procedure was implemented by using PROC PSMATCH in SAS [18]. In the two-tailed Wald test, a *p* < 0.05 was considered to indicate significance. Poisson regression models were used to compare overall and site-specific cancer incidence rates in patients with sarcopenia with those of the general population by estimating IRRs (95% Cis). We also applied the Kaplan–Meier estimator to calculate the cumulative incidence of cancer and overall survival in PS-matched patients with and without sarcopenia, and the differences between the sarcopenia and nonsarcopenia groups were determined by using the stratified log-rank test, so as to compare cancer incidence (stratified according to the matched sets) [19].

## 3. Results

### 3.1. PSM and Study Cohort

PSM yielded a final cohort of 77,608 patients (i.e., 15,527 and 62,081 in the sarcopenia and nonsarcopenia groups, respectively) who were eligible for further analysis; their characteristics are listed in Table 1. Age distribution was balanced between the two groups (Table 1). Furthermore, after head-to-head PSM, the between-group differences in sex, CCI Scores, diabetes, hypertension, hyperlipidemia, tuberculosis, pneumoconiosis, upper respiratory tract infection, hepatitis B, hepatitis C, liver cirrhosis, inflammatory bowel disease, familial adenomatous polyposis, urinary tract infection, Parkinson’s disease, child delivery, gum and periodontal disease, gastric or duodenal ulcer, sleep disorder, alcohol habits, cigarette smoking, income levels, and urbanization were nonsignificant. The cancer risk (before cancer diagnosis) in the sarcopenia group significantly differed from that in the nonsarcopenia group (*p* < 0.001; Table 1).

### 3.2. Cancer Risk Predictors after Multivariate Cox Regression Analysis

The results of multivariate Cox regression analysis indicated that the patients with sarcopenia had a higher cancer risk than did those without (Table 2). No significant differences were observed in explanatory variables (Table S1), except for male sex, old age (>65 years), diabetes, tuberculosis (TB), hepatitis B, hepatitis C, alcohol consumption, cigarette smoking, and low income level. The adjusted HR (aHR; 95% CI) for cancer risk in the sarcopenia group compared with the nonsarcopenia group was 1.277 (1.10 to 1.36; *p* < 0.001). The aHRs (95% CIs) for cancer risk in those aged 66–75, 76–85, and >85 years compared with those aged ≤65 years were 3.585 (3.13 to 6.08), 7.914 (7.10 to 11.79), and 9.292 (8.00 to 18.69), respectively (Table 2). The aHRs (95% CI) for cancer risk in patients who were male, had diabetes, had TB, had hepatitis B, had hepatitis C, consumed alcohol, smoked cigarettes, and had high income level (>NTD30,000/month) were 1.245 (1.21 to 1.29), 1.210 (1.15 to 1.27), 1.156 (1.02 to 1.31), 1.501 (1.37 to 1.64), 2.479 (1.03 to 5.96), 1.956 (1.22 to 2.19), 1.909 (1.25 to 2.97), and 0.790 (0.66 to 0.95), respectively; these data are based on a comparison with patients who were female, did not have diabetes, did not have TB, did not have hepatitis B, did not have hepatitis C, did not consume alcohol, did not smoke, and had a low income level, respectively.

### 3.3. Incidence Rates and IRRs of Different Cancer Types

We next calculated the significant adjusted IRRs of cancer risk on hepatocellular carcinoma (HCC), as well as colorectal, breast, head and neck, pancreas, gastric, esophageal, and ovarian cancers among the patients with or without sarcopenia diagnosis before cancer (Table 3). Prostate cancer risk did not differ significantly between the patients with and without sarcopenia (adjusted IRR, 1.13; 95% CI, 0.75 to 1.09). By contrast, compared with patients without sarcopenia, the patients with sarcopenia had adjusted IRRs (95% CI) of lung cancer 2.66 (1.15 to 2.90), HCC 1.84 (1.30 to 2.36), colorectal cancer 2.04 (1.77 to 2.30), breast cancer 1.56 (1.12 to 1.95), head and neck cancer 2.15 (1.44 to 2.53), pancreatic cancer 3.77 (1.79 to 4.01), gastric cancer 2.25 (1.54 to 3.23), esophageal cancer 3.38 (1.87 to 4.11), ovarian cancer 1.43 (1.10 to 2.29), and other cancers 1.86 (1.30 to 2.03), respectively (Table 3).

### 3.4. Cumulative Cancer Risks and Kaplan–Meier Curve for Overall Survival

**Figure 1:**
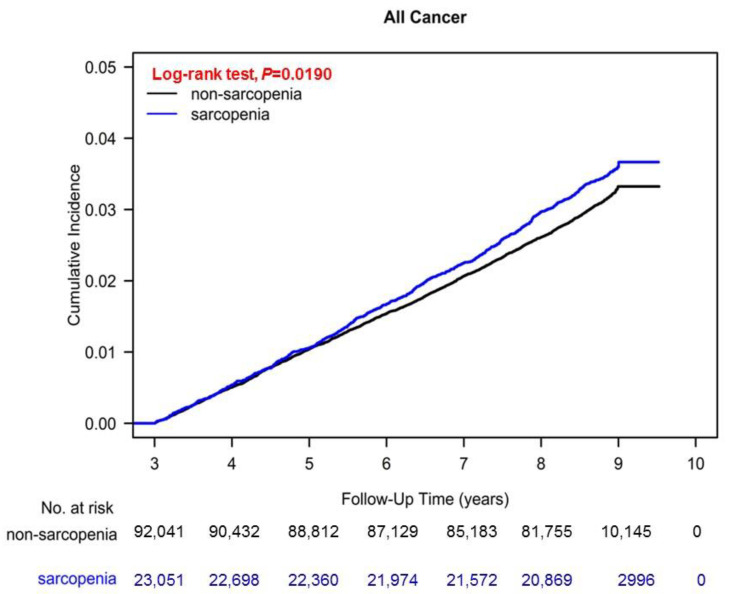
Cumulative cancer risks between PS-matched patients with and without sarcopenia.

**Figure 2:**
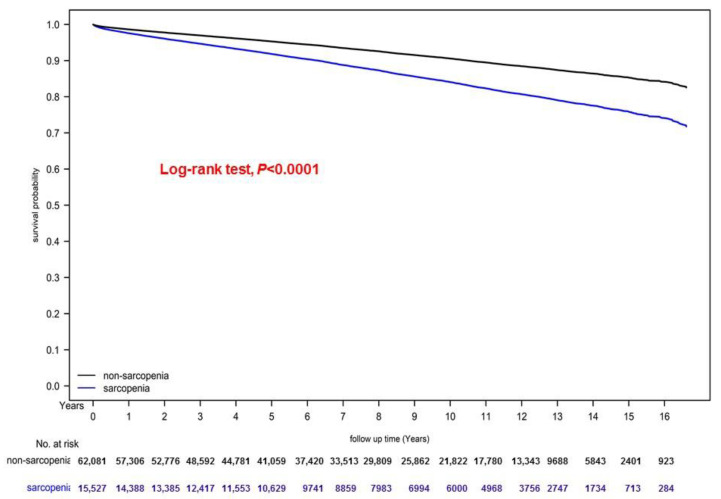
Kaplan–Meier curves for overall survival of patients with and without sarcopenia.

Figure 1 presents the cumulative risks of different cancer types between PS-matched patients with and without sarcopenia. As shown in Figure 1, the cumulative cancer risk was significantly higher in the sarcopenia group than in the nonsarcopenia group (*p* = 0.019). Moreover, the Kaplan–Meier curves indicated that overall survival was significantly lower in patients with sarcopenia than in those without (*p* < 0.001; Figure 2).

## 4. Discussion

Sarcopenia is a multifactorial syndrome resulting from inflammation and metabolic disorders; it can result from alterations in the endocrine function, activation of proinflammatory cytokines, reduction in the number of alpha motor units in the spinal cord, decreases in physical activity, and protein intake at suboptimal levels [20]. Highly inflammatory cytokines are negatively correlated with muscle strength and mass [21]. In general, sarcopenia is associated with high serum inflammatory parameters [22]. Chronic low-grade inflammation also contributes to losses in muscle mass, strength, and functionality—the main characteristics of sarcopenia [23]. In particular, chronic inflammation can cause DNA damage over time, resulting in cancer [24]. For example, chronic inflammatory bowel diseases, such as ulcerative colitis and Crohn disease, increases colon cancer risk [25]. Therefore [20], sarcopenia with higher serum inflammatory parameters may be an independent risk factor for cancer [20]. However, only a few studies have estimated the correlation between preexisting sarcopenia and cancer risk, even though many studies have demonstrated sarcopenia to be an independent poor prognostic factor for survival in patients with cancer [6,7,8]. The current study represents the first and the largest PSM-based comparative analysis for cancer risk in patients with and without sarcopenia. The results indicated a significant aHR for cancer and significant adjusted IRRs for HCC, as well as lung, colorectal, breast, head and neck, pancreatic, gastric, esophageal, and ovarian cancers, in patients with sarcopenia compared with those without sarcopenia.

A PSM-based design, such as that in the current study, can resolve this issue by maintaining a balance among the confounding factors of the case and control groups—all in the absence of bias. Moreover, PSM is currently the recommended standard tool for estimating the effects of covariates in studies where any potential bias may exist. Although the main advantage of the PSM methodology is the more precise estimation of the covariate effect, PSM cannot control for factors not accounted for in the model. Moreover, PSM is predicated on an explicit selection bias of those who could be matched; in other words, individuals who could not be matched are not part of the scope of inference.

We did not only use the latest criteria (ICD-10-CM code M62.84) from 2016 [15], but also included ICD-9-CM codes 728.2 and 728.9 [16] as sarcopenia and diagnosis of sarcopenia were verified by the professional specialists (such as rehabilitation, orthopedic, or family physician). Long-term follow-up time would be necessary for the time of cancers formation (named cancer incidence) after exposure of sarcopenia or other cancer risk factors. A short-term follow-up time with fast cancer incidence means that the association of carcinogens and cancer formation is weak. Cancers might have existed earlier but had not been screened and diagnosed. The time washout of sarcopenia and cancer incidence would be necessary. Therefore, we cannot analyzed only from 2016 onward.

In the current study, our multivariable Cox regression analysis results indicated that male sex, old age (>65 years), diabetes, tuberculosis (TB), hepatitis B, hepatitis C, alcohol consumption, cigarette smoking, and low income level are significant risk factors for cancer—corroborating the results of previous studies [26,27,28,29,30,31,32,33,34,35,36]. Age is the most crucial risk factor for a majority of cancers. According to the American National Cancer Institute, the median patient age at the time of a cancer diagnosis is 66 years [26]. Moreover, a majority (60%) of patients with cancer are aged 65 years or older, a quarter of new cancer cases are diagnosed at ages of 65–74 years [26], and the most common cancers occur more often in older patients [26]. Furthermore, diabetes is associated with an increased risks of liver, pancreas, endometrium, colon, rectal, breast, and bladder cancers [27]. TB may also increase lung cancer risk: TB causes substantial, prolonged pulmonary inflammation, which leads to host tissue damage, fibrosis, scar formation, genetic alterations, and, eventually, lung cancer [28]. Furthermore, TB has been found to be associated with significantly increased risks of non-Hodgkin’s lymphoma, as well as cervical, esophageal, ovarian, and breast cancers [29]. A chronic hepatitis B or hepatitis C virus infection can lead to liver cancer [30]. Moreover, hepatitis B virus infection has been noted to be associated with the risk of non-liver cancers, particularly those of the digestive system [31]. Heavy alcohol consumption significantly increases the risk of oral and pharyngeal cancer and esophageal squamous cell carcinoma by approximately 5-fold and that of laryngeal cancer by 2.5-fold; it also increases the risk of colorectal and breast cancers by 50% and that of pancreatic cancer by 30% [32]. Tobacco use is associated with many cancers, including lung, laryngeal, mouth, esophageal, throat, bladder, kidney, liver, stomach, pancreatic, colon, rectal, and cervical cancers, as well as acute myeloid leukemia [33]. Low income levels are also associated with the risk of some cancer, particularly breast, lung, and colorectal cancers [34,35,36]. In the current study, after most confounding factors were controlled for through PSM and adjusted by using the Cox regression model, sarcopenia became a significantly independent risk factor for cancers.

The current study is the first to indicate the hazard ratios for specific cancers risk in patients with preexisting sarcopenia. According to the values of the adjusted IRRs for various common cancers in this study, sarcopenia was found to contribute to pancreatic cancer in most cases, followed by esophageal cancer, lung cancer, gastric cancer, head and neck cancers, colorectal cancer, HCC, breast cancer, and, finally, ovarian cancer. Moreover, sarcopenia and prostate cancer demonstrated no significant relationship.

Sarcopenia has a high prevalence (≥50%) among patients with pancreatic, lung, or esophageal cancer, and it is associated with worse survival outcomes after surgery, radiotherapy, and chemotherapy [5]. However, no evidence suggesting that preexisting sarcopenia is a risk factor for pancreatic, lung, and esophageal cancers has been reported thus far; specifically, the cause–effect relationship between these cancers and sarcopenia (i.e., whether pancreatic, lung, or esophageal cancer induces sarcopenia or sarcopenia leads to pancreatic, lung, or esophageal cancer) remains unclear. Nevertheless, in the current study, we found that preexisting sarcopenia might be a risk factor for pancreatic, lung, or esophageal cancer. Kim et al. reported that approximately 41.7% of their patients with gastric cancer were diagnosed as having sarcopenia [37]. Our current results corroborate the authors’ observations: sarcopenia is associated with, and thus may be a novel risk factor for, gastric carcinogenesis [37]. Sarcopenia may also be associated with decreases in long-term survival in patients with head and neck cancer, colorectal cancer, HCC, breast cancer, and ovarian cancer [38]. Nevertheless, the present study is the first report that sarcopenia might be an independent risk factor for head and neck, colorectal, breast, and ovarian cancers.

Because sarcopenia is preventable and treatable, understanding the association of sarcopenia with cancer risk is essential [10]. In our study, sarcopenia is a significant cancer risk factor for lung, colorectal, breast, head and neck, pancreas, gastric, esophageal, and ovarian cancer, as well as hepatocellular carcinoma (Table 3). Aggressive treatments for sarcopenia, such as selective androgen receptor modulators or angiotensin-converting enzyme inhibitors, might be considered [20]. In addition, properly performed strength training can help to prevent or improve sarcopenia and osteoporosis [39]. In the near future, establishing correct a health policy for the alleviation of sarcopenia, leading to cancer-risk reduction, can have major future implications in the field of preventive medicine.

This study was the first and largest and long-term follow-up comparative cohort study to estimate the primary endpoint of cancer IRRs in patients with and without sarcopenia. The covariates between the two groups were homogenous, and any bias between the two groups was removed through PSM (Table 1). Only a few studies so far have estimated the risks of a wide variety of cancers in patients with sarcopenia. Nevertheless, the current study demonstrated that sarcopenia might be a significant risk factor for lung, colorectal, breast, head and neck, pancreas, gastric, esophageal, and ovarian cancers, as well as HCC.

This study has some limitations. First, because all patients with and without sarcopenia were enrolled from an Asian population, the corresponding ethnic susceptibilities in the non-Asian population remain unclear. However, although no evidence indicating a significant difference in the cancer risks between Asian and non-Asian populations has been reported, the current results should be cautiously extrapolated to non-Asian populations. Second, the diagnoses of all comorbid conditions were based on ICD-9-CM codes in this study. Nevertheless, the NHI Administration reviews charts and interviews of the beneficiaries in the NHIRD to verify the accuracy of the diagnoses, and it audits hospitals with outlier chargers or practices and subsequently heavily penalizes them if it identifies any malpractice or discrepancies. However, to obtain precise population specificity and disease occurrence data, a large-scale RCT carefully comparing patients diagnosed as having sarcopenia before cancer diagnosis with those without sarcopenia is warranted, but such RCTs may be difficult to execute. Third, the competing risk of mortality and cancer incidence might have been higher in the sarcopenia patients with higher mortality than in the patients without sarcopenia. In our study, the overall survival was poorer in the patients with sarcopenia (Figure 2); however, if the sarcopenia patients with poor survival would have survived, the overall cancer incidence would be increased in the sarcopenia group. Therefore, our conclusion that cancer risk is high in patients with sarcopenia would be only underestimated and could not be turned over. Finally, the NHIRD does not contain information regarding the patients’ dietary habits, which may be risk factors for cancer. Despite these limitations, the use of a nationwide population-based registry with detailed baseline information is a major strength of the current study. The NHIRD data are linked with Taiwan’s National Cause of Death Database; thus, in the current study, we could perform a lifelong follow-up for most patients. Considering the magnitude and statistical significance of the observed effects in the current study, the limitations are unlikely to have affected our conclusions.

## 5. Conclusions

Our results indicated that sarcopenia (sarcopenia, muscular wasting, disuse atrophy, and disorder) might be a significant cancer risk factor for HCC, as well as lung, colorectal, breast, head and neck, pancreas, gastric, esophageal, and ovarian cancers. Moreover, we noted that mortality is higher among patients with sarcopenia than among patients without sarcopenia—regardless of whether they develop cancer later. Our findings could be a highly valuable reference for establishing health policies. In general, early sarcopenia prevention and treatment may be valuable for not only improving the patients’ quality of life but also increasing their survival and reducing their cancer risks. For instance, physicians may encourage their patients to overcome sarcopenia more aggressively.

## Figures and Tables

**Table 1:** Characteristics of PS-matched patients with and without sarcopenia.

	Nonsarcopenia		Sarcopenia		SMD
	N = 62,081		N = 15,527		
	n	%	n	%	
Age (mean ± SD)	55.21 ± 21.35		57.55 ± 17.95		0.119
Age (y)					
≤65	37,348	60.16%	9449	60.86%	0.030
65–75	14,460	23.29%	3616	23.29%	
75–85	8499	13.69%	2042	13.15%	
>85	1774	2.86%	420	2.70%	
Sex					0.037
female	30,022	48.36%	7793	50.19%	
male	32,059	51.64%	7734	49.81%	
CCI Score (mean ± SD)	0.71 ± 1.11		0.75 ± 1.14		0.033
CCI Score					0.023
0	38,817	62.53%	9533	61.40%	
≥1	23,264	37.47%	5994	38.60%	
CCI					
Congestive heart failure	4304	6.93%	1023	6.59%	0.007
Dementia	1857	2.99%	505	3.25%	0.012
Chronic pulmonary disease	9642	15.53%	2438	15.70%	0.005
Rheumatic disease	660	1.06%	198	1.28%	0.026
Liver disease	8192	13.20%	2341	15.08%	0.021
Diabetes with complications	2491	4.01%	632	4.07%	0.015
Hemiplegia and paraplegia	0	0	0	0	0
Renal disease	2282	3.68%	550	3.54%	0.002
AIDS	27	0.04%	5	0.03%	0.001
Diabetes	10,906	17.57%	2748	17.70%	0.003
Hypertension	26,312	42.38%	6553	42.20%	0.004
Hyperlipidemia	13,090	21.09%	3453	22.24%	0.028
TB	1689	2.72%	445	2.87%	0.009
Pneumoconiosis	320	0.52%	79	0.51%	0.001
Upper respiratory tract infection	48,521	78.16%	12,321	79.35%	0.029
Hepatitis B	1767	2.85%	472	3.04%	0.011
Hepatitis C	58	0.09%	22	0.14%	0.014
Liver Cirrhosis	11,706	18.86%	3437	22.14%	0.081
Inflammatory bowel disease	1180	1.90%	321	2.07%	0.012
Familial adenomatous polyposis	751	1.21%	219	1.41%	0.018
Urinary tract infection	13,989	22.53%	3939	25.37%	0.066
Parkinson’s disease	1542	2.48%	424	2.73%	0.015
Child delivery	1116	1.80%	272	1.75%	0.003
Gum and periodontal disease	25,203	40.60%	6829	43.98%	0.069
Gastric or duodenal ulcer	21,648	34.87%	5567	35.85%	0.021
Sleep disorder	26,945	43.40%	7582	48.83%	0.109
Alcohol habits	22,439	36.14%	6274	40.41%	0.088
Cigarette smoking	10,366	16.70%	2714	17.48%	0.021
Income levels (NTD/month)					0.016
Low income	1018	1.64%	265	1.71%	
≤20,000	37,995	61.20%	9534	61.40%	
20,000–30,000	13,642	21.97%	3429	22.08%	
>30,000	9426	15.18%	2299	14.81%	
Urbanization					0.041
Rural	19,748	31.81%	5712	36.79%	
Urban	42,333	68.19%	9815	63.21%	
					*p*
Follow up time, y (mean ± SD)	7.65 ± 4.60		7.99 ± 4.66		0.481
Cancer					<0.001
No	57,619	92.81%	13,455	86.66%	
Yes	4462	7.19%	2072	13.34%	

Abbreviations: y, years; AIDS, acquired immune deficiency syndrome; CCI, Charlson comorbidity index; SD, standard deviation; SMD, standardized mean difference; NTD, New Taiwan Dollar; N, number; TB, tuberculosis.

**Table 2:** Univariable and Multivariable Cox Proportional Regression Model for Cancer Risk in PS-Matched Patients with and Without Sarcopenia. (Only show statistical significance).

	Crude HR (95% CI)		*p*	Adjusted HR * (95% CI)		*p*
Sarcopenia (ref.: nonsarcopenia)						
Yes	1.912	(1.78 to 2.05)	<0.0001	1.277	(1.10 to 1.36)	<0.0001
Sex (ref.: female)						
Male	1.217	(1.18 to 1.26)	<0.0001	1.245	(1.21 to 1.29)	<0.0001
Age (ref.: ≤65; y)						
65–75	3.861	(3.40 to 6.36)	<0.0001	3.585	(3.13 to 6.08)	<0.0001
75–85	7.402	(6.58 to 12.29)	<0.0001	7.914	(7.10 to 11.79)	<0.0001
>85	9.615	(8.27 to 20.06)	<0.0001	9.292	(8.00 to 18.69)	<0.0001
Diabetes (ref.: No)						
Yes	3.746	(3.59 to 3.91)	<0.0001	1.210	(1.15 to 1.27)	<0.0001
TB (ref.: No)						
Yes	2.994	(2.64 to 3.4)	<0.0001	1.156	(1.02 to 1.31)	0.0265
Hepatitis B (ref.: No)						
Yes	2.089	(1.91 to 2.28)	<0.0001	1.501	(1.37 to 1.64)	<0.0001
Hepatitis C (ref.: No)						
Yes	5.617	(2.35 to 13.43)	0.0001	2.479	(1.03 to 5.96)	0.0424
Alcohol habits (ref.: No)						
Yes	1.302	(1.26 to 1.35)	<0.0001	1.956	(1.22 to 2.19)	0.0124
Cigarette smoking (ref.: No)						
Yes	1.166	(1.10 to 1.24)	<0.0001	1.909	(1.25 to 2.97)	0.0023
Income (ref.: low income)						
≤20,000	0.911	(0.76 to 1.09)	0.3028	0.871	(0.73 to 1.04)	0.1271
20,000–30,000	0.922	(0.82 to 2.18)	0.3871	0.954	(0.8 to 1.14)	0.6030
>30,000	0.945	(0.90 to 1.37)	0.1381	0.790	(0.66 to 0.95)	0.0105

Abbreviations: y, years; NTD, New Taiwan Dollar; TB, tuberculosis; CI, confidence interval; HR, hazard ratio; ref., reference group. * All covariates presented in this table were adjusted.

**Table 3:** Incidence rates and IRRs of cancer in PS-matched patients with and without sarcopenia.

Cancer Types	Nonsarcopenia			Sarcopenia			IRRs * (95% CI)	*p*
	N = 62,081			N = 15,527				
	n	%	Incidence Rate (Per 1000 Person-y)	n	%	Incidence Rate (Per 1000 Person-y)		
Lung cancer	800	1.29%	7.27	504	3.25%	19.31	2.66 (1.15 to 2.90)	<0.0001
HCC	617	0.99%	6.78	256	1.65%	12.45	1.84 (1.30 to 2.36)	<0.0001
Colorectal cancer	650	1.05%	7.26	320	2.06%	14.81	2.04 (1.77 to 2.30)	<0.0001
Breast cancer	267	0.43%	3.00	102	0.66%	4.69	1.56 (1.12 to 1.95)	0.0002
Prostate cancer	355	0.57%	4.20	106	0.68%	4.73	1.13 (0.75 to 1.09)	0.1079
Head and Neck Cancer	169	0.27%	1.59	79	0.51%	3.42	2.15 (1.44 to 2.53)	<0.0001
Pancreatic cancer	65	0.10%	0.75	52	0.33%	2.83	3.77 (1.79 to 4.01)	<0.0001
Gastric cancer	212	0.34%	2.20	105	0.68%	4.96	2.25 (1.54 to 3.23)	<0.0001
Esophageal cancer	67	0.11%	0.58	43	0.28%	1.96	3.38 (1.87 to 4.11)	<0.0001
Ovarian cancer	47	0.08%	0.68	26	0.17%	0.97	1.43 (1.10 to 2.29)	0.0009
Others	1607	2.59%	17.32	747	4.81%	32.15	1.86 (1.30 to 2.03)	<0.0001

Abbreviations: y, year; IRR, incidence rate ratio; HCC, hepatocellular carcinoma. * All covariates presented in this table were adjusted.

## Data Availability

The datasets supporting the study’s conclusions are included within this manuscript and Supplementary Materials.

## Supplementary Materials

The following supporting information can be downloaded at: https://www.mdpi.com/article/10.3390/nu14091910/s1, Table S1: Univariable and multivariable Cox proportional regression model for cancer risk in PS-matched patients with and without sarcopenia (shows non-statistical significance).
