# Supplementary material for: Sarcopenia as an Independent Risk Factor for Specific Cancers: A Propensity Score-Matched Asian Population-Based Cohort Study
Source: Nutrients. 2022 May 2;14(9):1910. doi: 10.3390/nu14091910 (PMC9105218; doi:10.3390/nu14091910)
Supplement: Supplementary file 1 [file nutrients-14-01910-s001.zip › nutrients-1687125-supplementary.pdf]

Supplementary Table S1. Univariable and multivariable Cox proportional regression model for cancer risk in PS-matched patients with and without sarcopenia (shows non-statistical significance).

	**Crude HR (95% CI)**		***p***	**Adjusted HR* (95% CI)**		***p***
CCI score (ref.: CCI = 0)						
≥1	1.519	(1.43 to 2.61)	0.1565	1.323	(0.84 to 1.34)	0.2185
**Hyperlipidemia** (ref.: No)						
Yes	1.04	(0.72 to 3.06)	0.1741	1.024	(0.88 to 1.97)	0.2935
Hypertension (ref.: No)						
Yes	1.159	(0.92 to 2.31)	0.6351	1.075	(0.93 to 1.12)	0.1874
Pneumoconiosis (ref.: No)						
Yes	1.464	(0.62 to 3.51)	0.6430	1.394	(0.73 to 1.72)	0.2642
Upper respiratory tract infection (ref.: No)						
Yes	1.085	(0.76 to 1.81)	0.3752	1.017	(0.88 to 1.95)	0.2625
Liver cirrhosis (ref.: No)						
Yes	1.075	(1.01 to 2.17)	<0.0151	1.124	(0.91 to 1.41)	0.2361
Inflammatory bowel disease						
	1.532	(0.80 to 2.36)	0.4583	1.462	(0.84 to 1.69)	0.6215
Familial adenomatous polyposis						
	1.410	(0.78 to 2.14)	0.3465	1.340	(0.82 to 1.71)	0.5038
Urinary tract infection						
	1.392	(0.77 to 2.08)	0.2888	1.278	(0.81 to 1.69)	0.4916
Parkinson's disease (ref.: No)						
Yes	1.643	(1.12 to 4.25)	<0.0001	1.579	(0.84 to 1.74)	.7923
Child Delivery (ref.: No)						
Yes	0.565	(0.51 to 0.63)	<0.0001	0.871	(0.65 to 1.44)	.4297
Gum and periodontal disease (ref.: No)						
	1.311	(0.89 to 2.22)	.3698	1.301	(0.71 to 1.71)	.4402
Gastric or duodenal ulcer (ref.: No)						
	1.103	(0.78 to 1.80)	.2863	1.071	(0.68 to 1.74)	.3103
Sleep disorder (ref.: No)						
	1.089	(0.66 to 1.74)	.1652	1.090	(0.65 to 1.61)	.2892
Urbanization (ref.: rural)						
Urban	1.210	(0.83 to 1.44)	.4439	1.534	(0.92 to 2.07)	.5761

Abbreviations: CCI, Charlson comorbidity index; CI, confidence interval; HR, hazard ratio; ref., reference group. *All covariates presented in Table 2 were adjusted.
